# Maintained inspiratory activity during proportional assist ventilation in surfactant-depleted cats early after surfactant instillation: phrenic nerve and pulmonary stretch receptor activity

**DOI:** 10.1186/1465-9921-7-38

**Published:** 2006-03-10

**Authors:** Richard Sindelar, Esther Rieger-Fackeldey, Anders Jonzon, Peter Schaller, Andreas Schulze, Gunnar Sedin

**Affiliations:** 1 Department of Women's and Children's Health and Department of Neuroscience, Physiology, Uppsala University, Uppsala, Sweden; 2 Department of Obstetrics and Gynecology, Division of Neonatology, Klinikum Grosshadern, Ludwig Maximilian University, Munich, Germany; 3 Children's Hospital, Carl Gustav Carus Faculty of Medicine, University of Dresden, Dresden, Germany

## Abstract

**Background:**

Inspiratory activity is a prerequisite for successful application of patient triggered ventilation such as proportional assist ventilation (PAV). It has recently been reported that surfactant instillation increases the activity of slowly adapting pulmonary stretch receptors (PSRs) followed by a shorter inspiratory time (Sindelar et al, J Appl Physiol, 2005 [Epub ahead of print]). Changes in lung mechanics, as observed in preterm infants with respiratory distress syndrome and after surfactant treatment, might therefore influence the inspiratory activity when applying PAV early after surfactant treatment.

**Objective:**

To investigate the regulation of breathing and ventilatory response in surfactant-depleted young cats during PAV and during continuous positive airway pressure (CPAP) early after surfactant instillation in relation to phrenic nerve activity (PNA) and the activity of PSRs.

**Methods:**

Seven anesthetized, endotracheally intubated young cats were exposed to periods of CPAP and PAV with the same end-expiratory pressure (0.2–0.5 kPa) before and after lung lavage and after surfactant instillation. PAV was set to compensate for 75% of the lung elastic recoil.

**Results:**

Tidal volume and respiratory rate were higher with lower PaCO_2 _and higher PaO_2 _during PAV than during CPAP both before and after surfactant instillation (p < 0.05; both conditions). As an indicator of breathing effort, esophageal deflection pressure and PNA were lower during PAV than during CPAP in both conditions (p < 0.02). Peak PSR activity was higher and occurred earlier during PAV than during CPAP (p < 0.01), and correlated linearly with PNA duration in all conditions studied (p < 0.001). The inspiratory time decreased as tidal volume increased when CPAP was changed to PAV, with the highest correlation observed after surfactant instillation (r = -0.769). No apneic periods could be observed.

**Conclusion:**

PSR activity and the control of breathing are maintained during PAV in surfactant-depleted cats early after surfactant instillation, with a higher ventilatory response and a lower breathing effort than during CPAP.

## Background

Proportional assist ventilation (PAV) is a new mode of assisted ventilation wherein the applied airway pressure is servo-controlled continuously throughout spontaneous inspiration, changing in proportion to the patient's breathing effort and allowing the patient to control the extent and timing of lung inflation [1-3]. The ventilator can thus be set to unload a certain proportion of the elastic forces needed to inflate a certain volume at a given level of lung compliance, i.e. elastic unloading [4]. Additionally, resistive unloading can be applied in combination with elastic unloading, reducing the resistive work of breathing during inspiration and expiration [5].

In comparison with spontaneous breathing on continuous positive airway pressure (CPAP), PAV has been reported to increase the tidal volume and decrease arterial PaCO_2 _in cats with severe respiratory failure [6]. The same study showed that phrenic nerve activity (PNA) was lower during PAV than during CPAP, in terms of the amplitude and duration of the integrated PNA [6]. In a clinical study of low birth weight infants with mild respiratory distress syndrome (RDS), at a postnatal age of >24 hours, PAV maintained gas exchange with lower transpulmonary pressures compared with assist control ventilation and intermittent mandatory ventilation [7].

The activity of slowly adapting pulmonary stretch receptors (PSRs) has been extensively studied and is believed to modify both the depth and rate of breathing [8-12], and to play an important role in the Hering-Breuer inspiratory inhibitory reflex [13]. Studies of infants with RDS and preterm infants have shown that this reflex, elicited by the end-inspiratory occlusion technique, seems to be stronger in these infants than in healthy infants born at term [14,15]. In addition, PSRs have been shown to increase their activity after instillation of surfactant in spontaneously breathing surfactant-depleted young cats, accompanied by a shorter inspiratory time and a lower inspiratory to expiratory time ratio [16]. These findings could have implications for successful application of PAV in infants with RDS as the control of breathing might be influenced by apnoea of prematurity early after surfactant administration of preterm infants with RDS [17].

The non-compliant lung requires a larger amount of elastic unloading, i.e., a gradual increase in airway pressure during inspiration, in order to attain a close to normal compliance of the combined lung-respirator system. Although the applied airway pressure during PAV is proportional to the ongoing inspiratory effort, the increased PSR activity during recovery from RDS after surfactant instillation [16] might elicit an earlier termination or abolishment of the inspiratory activity. No study has been focused on the use of PAV early after instillation of surfactant.

The aim of this study was therefore to investigate the control of breathing and the breathing pattern during PAV and CPAP in surfactant-depleted young cats early after surfactant instillation, with special respect to PNA and PSR activity.

## Methods

### General

Seven young cats with a mean body weight of 3.22 ± 0.59 kg (± S.D.) were anaesthetised with chloroform, intubated endotracheally, and connected to an infant ventilator (Stephanie^®^, F. Stephan Biomedical Inc., Gackenbach, Germany), which was set on controlled mechanical ventilation during the surgical procedures. In addition to conventional ventilation and CPAP, this ventilator provides negative and positive ventilator resistance and compliance [1]. The ventilator uses pressure-control feedback technology to generate the different airway pressure patterns. The feedback sampling rate of the system is >15 Hz, and the corresponding time constant is <10 ms. Since the respiratory rates in cats are much lower than this feedback sampling rate (<0.3 Hz; maximum of 25 breaths/min in our study), resistive and elastic unloading could be generated with a high degree of accuracy in this study.

The right femoral vein and artery were dissected and catheters were inserted so that their tips were located in the thorax. The venous catheter was used for maintenance of anesthesia with intravenous administration of 7.2 g/L d-chloralose (E. Merck AG, Darmstadt, Germany; initial dose 10 mL/kg, additional doses of 2.0–2.5 mL/kg/h). A mixture of 10% glucose (two-thirds) and 5% bicarbonate (one-third) was given through the same line at a rate of 6.4 mL/h. Arterial blood gases and pH were analyzed with an automatic acid base analyzer (ABL 300^®^, Radiometer Corp., Copenhagen, Denmark). Care was taken to maintain a normal body temperature.

A pretracheal midline incision was made and a ligature was tied around the trachea in order to prevent leakage around the tube. An 8 French catheter with an esophageal balloon (40 × 7.5 mm; flat frequency response up to 5 Hz) was advanced into the lower part of the esophagus for recording of pressure [18], and a ligature was then tied gently around the esophagus.

The phrenic nerve and the vagal nerve were exposed, each on either side of the neck. A small portion of the vagal nerve was gently dissected into thin filaments, thereby leaving the major part of the nerve intact. The filaments were placed on a single platinum electrode and their impulse activity was recorded and analyzed until a signal from a single slowly adapting pulmonary stretch receptor was recognized by its characteristic pattern of discharge during the ventilatory cycle and its slowly adapting activity during maintained inflation [19,20]. A reference electrode was placed in the nearby connective tissue. The nerves were immersed in mineral oil to prevent drying and for electrical insulation.

### Measurements and recordings

The arterial blood pressure and heart rate were measured continuously with a transducer (Druck Ltd. Transducer, Leicestershire, UK). Airflow was measured with a pneumotachograph head (resistance 1.1 kPa/L/s at a flow of 5 L/min; dead space 0.9 mL) at the ETT [21]. Esophageal pressure and airway pressure was measured with pressure transducers (Druck Ltd. Transducer, Leicestershire, UK).

PNA was amplified, filtered and rectified with a Neurolog system (Digitimer Research Instrumentation Inc., Welwyn Garden City, Hertfordshire, UK; preamplifier NL 103, AC amplifier NL 105, filters NL 115, spike trigger NL 200). The rectified nerve signal was integrated by a resistance-capacitance low-pass filter with a leak (time constant 250 ms), providing a moving time average of PNA [22,23]. The signals from PSRs were amplified with the same system. Spike amplitudes were fed to a spike generator to produce spikes of uniform duration (0.5 ms) and amplitude (Digitimer D 130^® ^and Spike Trigger NL 200, Digitimer Research Instrumentation Inc., Welwyn Garden City, Hertfordshire, UK). All recorded signals were digitized and recorded online by a data acquisition system (Windaq Data Acquisition^®^, Dataq Instruments Corp., Austin, USA).

### Protocol

The experiments were performed at the Biomedical Centre of Uppsala University and the protocol was approved by the Uppsala University Animal Research Ethics Board (D:no C 217/94; C 130/97).

During the preparatory and surgical procedures the cats were normoventilated within physiological ranges of pH and PaCO_2_. To achieve surfactant depletion similar to that in RDS, lung lavage was performed 7–8 times through the endotracheal tube with saline (30 mL/kg) heated to +37.5°C [24]. After 30 minutes of stabilization, the cats were allowed to breathe spontaneously on CPAP and PAV. Following a period of mechanical ventilation, the cats received an instillation of porcine surfactant (Curosurf^® ^100 mg/kg) through the endotracheal tube, and after 10 minutes of stabilization they were allowed to breathe spontaneously on CPAP and PAV.

Data from 10 to 20 consecutive breaths were recorded at the end of a 3-min stabilization period with CPAP or PAV before and after lung lavage and after surfactant instillation. CPAP was applied before and after PAV during each lung condition. The same end-expiratory pressure was set during PAV as during CPAP (0.2 kPa before lung lavage and 0.5 kPa both after lung lavage and after instillation of surfactant). Resistive unloading was set to compensate for the endotracheal tube resistance during inspiration only (2.0 kPa/L/s), and elastic unloading was set to compensate for 75 % of the elastic recoil of the respiratory system as calculated from lung compliance provided by measurements made prior to switching to PAV.

Recordings of CPAP and PAV were followed by blood gas measurements. Lung compliance was determined before each change of ventilatory mode and change of lung condition.

### Analysis of results

The Windaq Analysis Software^® ^(Dataq Instruments Corp., Austin, USA) was used to review and analyze all the acquired signals. The airflow signal was integrated to tidal volume.

The PSRs were classified as low-threshold receptors if they discharged throughout the breathing cycle and as high-threshold receptors if they discharged during inflation only [25].

*Transpulmonary pressure *was calculated as the difference between airway pressure and esophageal pressure. The *maximal esophageal pressure deflection *was calculated as the difference between the end-expiratory and the lowest esophageal pressure in each respiratory cycle. *Lung compliance *was calculated as tidal volume divided by the transpulmonary difference between the beginning of inspiration and the end-inspiration when no gas was flowing.

The *instantaneous impulse frequency of PSR activity (PSR f*_imp_) was calculated from the time interval between two consecutive spikes. The *peak PSR f*_imp _and the *time to peak PSR f*_imp _during each respiratory cycle were calculated.

The start of *the integrated phrenic nerve burst *coincided with the start of inspiratory airflow during spontaneous breathing. *The amplitude of the integrated PNA *was used as a measure of the total number of impulses in the phrenic nerve burst. *The mean impulse frequency of the PNA (mean PNA) *was calculated by dividing the amplitude of the integrated PNA by *the duration of the phrenic nerve burst *[22,23,26].

*Inspiratory time, expiratory time *and *respiratory rate *were calculated from the airflow recordings. *Inspiratory to expiratory time ratio (I:E ratio) *was calculated by dividing inspiratory time by expiratory time.

As an index of respiratory pattern variability, *the coefficient of variation (C.V.) *was calculated as the standard deviation (S.D.) of consecutive pairs of breaths divided by their mean value [16,27].

All 7 cats completed the entire protocol. One single unit of PSR was studied in each cat. Because of technical problems in maintaining the same nerve fiber from a single unit of PSR throughout the two interventions, 5 single-unit PSRs were studied before and after lung lavage and after surfactant instillation, and 2 single-unit PSRs were studied after lung lavage and after surfactant instillation. Of the 7 receptors studied, 4 were defined as high-threshold receptors and 3 as low-threshold receptors.

### Statistics

Repeated-measures analysis of variance was used to test for differences between data collected during CPAP and during PAV. Student's *t-test *for two-sided paired observations was applied whenever a difference was detected by analysis of variance, and differences were considered significant at *p *< 0.05.

## Results

**Figure 1:**
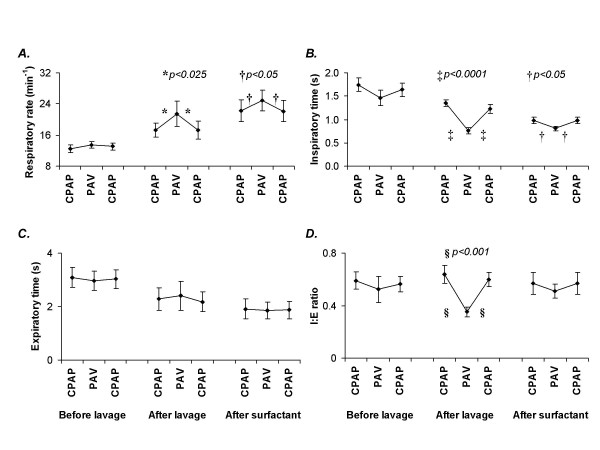
Respiratory rate (*A*), inspiratory time (*B*), expiratory time (*C*), and I:E ratio (*D*) during the two baseline periods of CPAP before and after PAV, and during PAV. Data are presented for the periods before and after lung lavage and after instillation of surfactant. Significant differences between PAV and the preceding and succeeding CPAP are marked with *, †, ‡ or § and their respective *p values *are presented. Means ± standard errors of the mean are shown.

There were no differences between the values for the measured variables obtained during CPAP before PAV and those obtained during CPAP after PAV in the different sequences of this study. Therefore only values obtained during the first period of CPAP are presented in the Result section, except in Figure 1.

### Lung mechanics and arterial blood gases

*Lung compliance *decreased by 63% after lung lavage and increased by 20% after instillation of surfactant (Table 1). In cats with *normal lungs*, arterial PaO_2 _was higher, while esophageal deflection pressure was 28% lower, during PAV than during CPAP (*p *< 0.01) (Table 1).

**Table 1:** Comparison of arterial blood gases, inspired oxygen fraction (F_i_O_2_), tidal volume (V_T_), esophageal deflection pressure (Δ P_eso_), transpulmonary pressure (P_tp_), and lung compliance (C_L_) between CPAP and PAV before and after lung lavage and after instillation of surfactant.

		**CPAP**	**PAV**	***CPAP-PAV p (ANOVA)***
		***Mean *± *S.D*.**	***Mean *± *S.D***.	
**Before lung lavage**	**pH**	7.31 ± 0.04	7.33 ± 0.03	*NS*
	**PaCO_2_, kPa**	5.47 ± 0.80	5.20 ± 0.80	*NS*
	**PaO_2_, kPa**	11.20 ± 0.80	12.40 ± 0.67	<*0.001*
	**F_i_O_2_**	0.21 ± 0.00	0.21 ± 0.0	*NS*
	**V_T_, mL**	36 ± 10	38 ± 6	*NS*
	**ΔP_eso_, kPa**	0.32 ± 0.09	0.18 ± 0.08	<*0.02*
	**P_tp_, kPa**	0.41 ± 0.12	0.44 ± 0.11	*NS*
	**C_L_, mL/cm H_2_O**	6.43 ± 1.96	6.43 ± 1.96	*NS*

**After lung lavage**	**pH**	7.24 ± 0.08 ^a^	7.29 ± 0.10	<*0.05*
	**PaCO_2_, kPa**	6.93 ± 1.73 ^a^	5.87 ± 1.60 ^a^	<*0.03*
	**PaO_2_, kPa**	8.27 ± 2.67 ^a^	9.47 ± 2.13 ^a^	<*0.01*
	**F_i_O_2_**	0.77 ± 0.18 ^a^	0.75 ± 0.20 ^a^	*NS*
	**V_T_, mL**	25 ± 9 ^a^	46 ± 28	<*0.02*
	**ΔP_eso_, kPa**	0.86 ± 0.36 ^a^	0.27 ± 0.21 ^a^	<*0.02*
	**P_tp_, kPa**	1.34 ± 0.53 ^a^	1.83 ± 0.57 ^a^	*p *<*0.0001*
	**C_L_, mL/cm H_2_O**	2.59 ± 0.76 ^a^	2.59 ± 0.76 ^a^	*NS*

**After surfactant instillation**	**pH**	7.22 ± 0.08	7.29 ± 0.11	<*0.01*
	**PaCO_2_, kPa**	7.73 ± 1.60	6.13 ± 2.00	<*0.01*
	**PaO_2_, kPa**	9.47 ± 2.40 ^b^	11.47 ± 2.53 ^b^	<*0.05*
	**F_i_O_2_**	0.76 ± 0.29	0.76 ± 0.29	*NS*
	**V_T_, mL**	25 ± 7	48 ± 16	<*0.01*
	**ΔP_eso_, kPa**	0.56 ± 0.31 ^b^	0.22 ± 0.09 ^b^	<*0.02*
	**P_tp_, kPa**	1.11 ± 0.49 ^b^	1.62 ± 0.25 ^b^	*p *<*0.0001*
	**C_L_, mL/cm H_2_O**	3.10 ± 0.61 ^b^	3.10 ± 0.61 ^b^	*NS*

^a ^significant difference between pre- and post-lavage values

^b^significant difference between post-lavage value and value after surfactant instillation

^a,b ^p < 0.05; ANOVA; two-tailed paired Student's t-test.

NS, non-significant; S.D., standard deviation.

*After lung lavage*, arterial PaCO_2 _was lower during PAV than during CPAP, which was explained by an 84% higher tidal volume and an 18% higher respiratory rate during PAV than during CPAP (Table 1) (Fig. 1, *panel A*). After lung lavage esophageal deflection pressure was 51% lower during PAV than during CPAP. The over-all effects of lung lavage was a reduction in PaO_2 _both during PAV and during CPAP as compared to the pre-lavage values, with a concomitant increase in inspired fraction of oxygen from 0.21 to 0.76 (Table 1).

*After instillation of surfactant*, arterial pH, tidal volume and respiratory rate remained higher and PaCO_2 _lower during PAV than during CPAP (*p *< 0.01) (Table 1) (Fig. 1, *panel A*). The most significant changes following instillation of surfactant, during both PAV and CPAP, were a higher PaO_2 _and a lower esophageal deflection pressure than before this instillation. However, esophageal deflection pressure was still 64% lower during PAV than during CPAP after instillation of surfactant (Table 1).

### Breathing pattern

In general, no apnoeic periods were detected during PAV in surfactant-depleted cats or in cats after instillation of surfactant.

*Before lung lavage*, no differences were observed between the respiratory rate, inspiratory time and expiratory time recorded during PAV and those recorded during CPAP (Fig. 1, *panels A-C*).

*After lung lavage*, respiratory rate was higher than before lung lavage during both PAV and CPAP (*p *< 0.05). During CPAP this difference was due to shortening of both inspiratory time and expiratory time (*p *< 0.01 and *p *< 0.04, respectively), but during PAV it was mainly due to shortening of inspiratory time (*p *< 0.01) (Fig. 1, *panels A-C*). Respiratory rate was higher and inspiratory time was shorter during PAV than during CPAP after lung lavage (*p *< 0.05 and *p *< 0.0001, respectively) (Fig. 1, *panels A and B*). These differences in inspiratory time and expiratory time between PAV and CPAP resulted in a lower I:E ratio during PAV than during CPAP after lung lavage (0.35 ± 0.08 vs. 0.63 ± 0.15; *p *< 0.03; ± S.D.) (Fig. 1, *panel D*).

During CPAP, respiratory rate was higher *after *than before *instillation of surfactant *(*p *< 0.01), while no corresponding change was seen during PAV. The higher respiratory rate during CPAP was due to shortening of both inspiratory time and expiratory time (*p *< 0.005 and *p *< 0.02, respectively) (Fig. 1, *panels A-C*), resulting in the same I:E ratio after as before instillation of surfactant. Although the I:E ratio did not differ between PAV and CPAP after instillation of surfactant, respiratory rate still remained higher during PAV than during CPAP (*p *< 0.05) (Fig. 1, *panels A and D*).

Respiratory rate, inspiratory time and expiratory time showed generally low C.V.s. Only after lung lavage was a difference in variability detected between CPAP and PAV, with a higher C.V. for respiratory rate during PAV than during CPAP (6.1 ± 1.6 vs. 2.6 ± 1.1 %; *p *< 0.01), as a result of a higher C.V. for expiratory time (7.5 ± 4.1 vs. 2.6 ± 1.4 %; *p *< 0.02). After instillation of surfactant there was no difference between CPAP and PAV as to the variability of these parameters.

### Phrenic nerve activity

*Before lung lavage *there was no difference in PNA between PAV and CPAP (Table 2).

**Table 2:** Slowly adapting pulmonary stretch receptor (PSR) activity and phrenic nerve activity (PNA) during CPAP and PAV before and after lung lavage and after instillation of surfactant.

		**CPAP**	**PAV**	***CPAP-PAV p (ANOVA)***
		***Mean *± *S.D***.	***Mean *± *S.D***.	
**Before lung lavage**	Peak PSR *f*_imp_, impulses*sec^-1^	64 ± 19	67 ± 20	*NS*
	**Time to peak PSR activity, sec**	1.48 ± 0.34	1.35 ± 0.10	*NS*
	PSR *f*_imp _per P_tp_, impulses*sec^-1^*kPa^-1^	1.78 ± 0.33	1.53 ± 0.25	*NS*
	**PNA duration, sec**	1.80 ± 0.38	1.64 ± 0.31	*NS*
	**PNA amplitude, AU**	0.58 ± 0.50	0.49 ± 0.34	*NS*
	**Mean PNA, AU/sec**	0.38 ± 0.42	0.34 ± 0.30	*NS*

**After lung lavage**	Peak PSR *f*_imp_, impulses*sec^-1^	45 ± 37 ^a^	88 ± 33 ^a^	*p *<*0.0001*
	**Time to peak PSR activity, sec**	1.18 ± 0.27 ^a^	0.78 ± 0.22 ^a^	*p *<*0.0001*
	PSR *f*_imp _per P_tp_, impulses*sec^-1^*kPa^-1^	0.42 ± 0.29 ^a^	0.46 ± 0.19 ^a^	*NS*
	**PNA duration, sec**	1.38 ± 0.22 ^a^	1.10 ± 0.48^a^	*p *<*0.05*
	**PNA amplitude, AU**	0.68 ± 0.51	0.46 ± 0.40	*p *<*0.03*
	**Mean PNA, AU/sec**	0.53 ± 0.48 ^a^	0.52 ± 0.58	*NS*

**After surfactant instillation**	Peak PSR *f*_imp_, impulses*sec^-1^	53 ± 36	82 ± 17	*p *<*0.0001*
	**Time to peak PSR activity, sec**	1.00 ± 0.16 ^b^	0.80 ± 0.09	*p *<*0.0001*
	PSR *f*_imp _per P_tp_, impulses*sec^-1^*kPa^-1^	0.59 ± 0.25 ^b^	0.55 ± 0.11 ^b^	*NS*
	**PNA duration, sec**	0.97 ± 0.24 ^b^	0.91 ± 0.09	*p *<*0.01*
	**PNA amplitude, AU**	0.75 ± 0.55	0.61 ± 0.64	*NS*
	**Mean PNA, AU/sec**	0.81 ± 0.69	0.67 ± 0.71 ^b^	*p *<*0.01*

^a ^significant difference between pre- and post-lavage values

^b ^significant difference between post-lavage value and value after surfactant instillation

^a,b ^p < 0.05. ANOVA; two-tailed paired Student's t-test. S.D., standard deviation; NS, non-significant; AU, arbitrary units; PSR *f*_imp_, instantaneous impulse frequency of PSR; P_tp_, transpulmonary pressure

**Figure 2:**
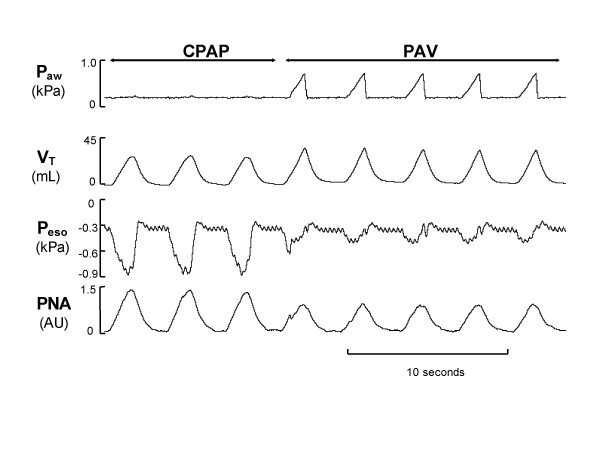
Airway pressure (*P*_*aw*_), tidal volume (*V*_*T*_), esophageal pressure (*P*_*eso*_) and phrenic nerve activity (*PNA*) during CPAP and PAV after lung lavage. Note the immediate decrease in PNA and the decrease in esophageal pressure on transition from CPAP to PAV, showing the combined breathing effort and elastic unloading during PAV. *AU*, arbitrary units.

Both *after lung lavage and after instillation of surfactant*, PNA was lower during PAV than during CPAP (Table 2). The decrease in PNA amplitude and duration during PAV, occurring concomitantly with the decrease in esophageal pressure and the increase in tidal volume, was immediate when CPAP was switched to PAV, as observed in a recording made after lung lavage (Fig. 2).

### Slowly adapting pulmonary stretch receptor activity

All receptors maintained their characteristic of being a high-threshold or a low-threshold receptor after each intervention.

*Before lung lavage*, there was no difference in peak PSR *f*_imp _between PAV and CPAP, although the same tidal volume was attained with a lower esophageal deflection pressure during PAV than during CPAP (Tables 1 and 2).

**Figure 3:**
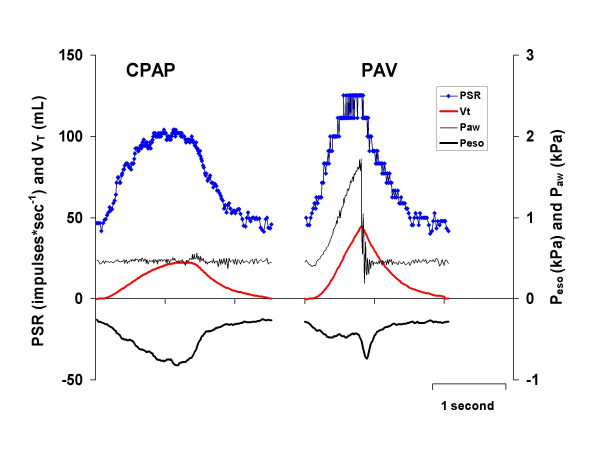
Recordings of P_*aw*_, V_*T*_, P_*eso*_, and PSR *f*_imp _during CPAP and PAV after lung lavage. Note the shorter time to peak PSR *f*_imp _and the higher peak PSR *f*_imp _during PAV than during CPAP, giving a shorter inspiratory time.

*After lung lavage *and *after instillation of surfactant*, the peak PSR *f*_imp _and transpulmonary pressure were higher during PAV than during CPAP (Tables 1 and 2). Also, the time to peak PSR *f*_imp _was shorter during PAV than during CPAP both after lung lavage (Table 2; Fig. 3), and after surfactant instillation (Table 2)

PSR *f*_imp _in relation to transpulmonary pressure decreased after lung lavage (*p *< 0.001) and increased after instillation of surfactant (*p *< 0.01) both during CPAP and PAV (Table 2). There were no differences in PSR *f*_imp _in relation to transpulmonary pressure between CPAP and PAV either before or after lung lavage or after instillation of surfactant (Table 2).

### Simultaneous changes in PSR *f*_imp_ and PNA during PAV

**Figure 4:**
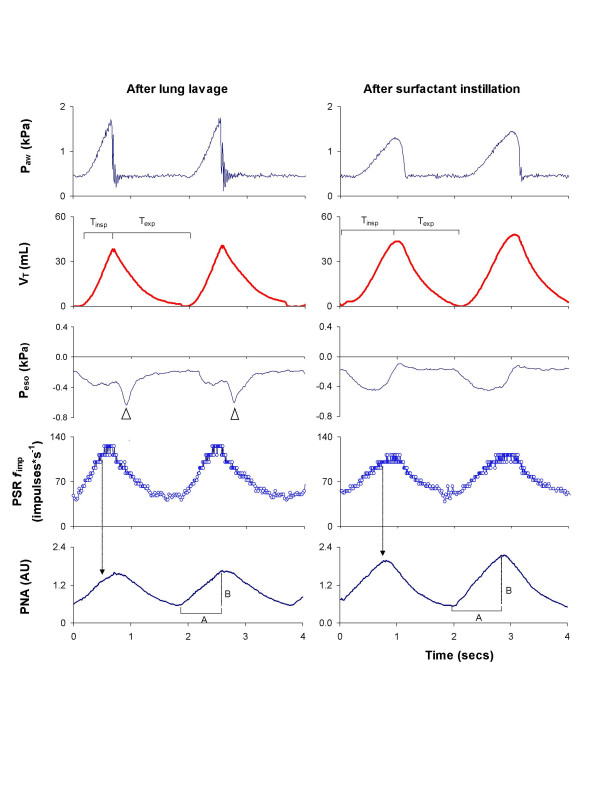
Recordings of P_*aw*_, V_*T*_, P_*eso*_, PSR *f*_imp_, and PNA during PAV after lung lavage and after instillation of surfactant. After lung lavage, peak PSR *f*_imp _was reached earlier than peak PNA amplitude, resulting in a lower I:E ratio than after instillation of surfactant. Peak PSR was lower after than before surfactant instillation, giving a higher PNA amplitude and longer PNA duration. The changes in esophageal pressure did not alter PSR *f*_imp_. Inspiratory time (*T*_*insp*_) and expiratory time (*T*_*exp*_) are marked in the recordings of V_*T*_. Black arrows point to the concomitant change in PNA when peak PSR *f*_imp _was reached. The duration (*A*) and amplitude (*B*) of PNA are indicated in the recordings of PNA. Note the late rebound of esophageal pressure after lung lavage (white arrows; please see further in the text for explanation).

**Figure 5:**
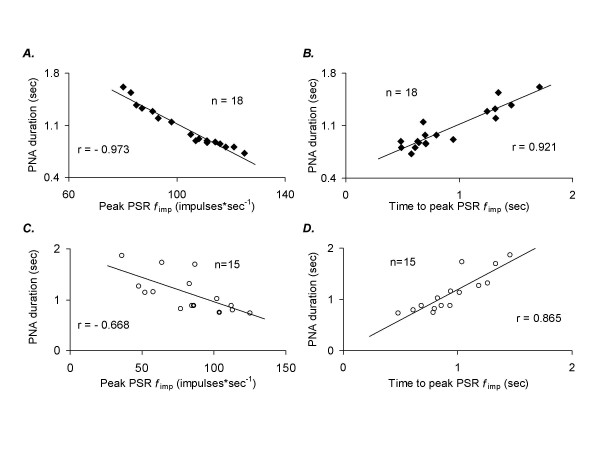
Linear correlation between peak PSR *f*_imp _and PNA duration (*A and C*), and between time to peak PSR *f*_imp _and PNA duration (*B and D*), during PAV before and after lung lavage, and after instillation of surfactant. A and B present recordings from one single-unit receptor (6 breaths per setting) in one cat; C and D present the mean values of recordings from 5 single-unit receptors from 5 cats that completed the entire protocol (one single-unit receptor per cat; see end of Methods). Note the inverse correlation between peak PSR *f*_imp _and PNA duration. For both correlations, *p *< 0001 (*panels A and B*) and *p *< 0.01 (*panels C and D*) *r*, correlation coefficient.

One example illustrating the timing of PSR *f*_imp _during PAV after lung lavage and after surfactant instillation in comparison to changes in esophageal pressure, tidal volume and PNA, is presented in Figure 4. During PAV before and after lung lavage and after surfactant instillation, there was a high linear correlation between peak PSR *f*_imp _and PNA duration in each individual cat (Fig. 5, *panel A; p *< 0.001) (range for all cats r = -0.965 to -0.986), as well as between time to peak PSR *f*_imp _and PNA duration (Fig. 5, *panel B; p *< 0.001) (range for all cats r = 0.914 to 0.933). Partly irrespective of interindividual differences in PSR *f*_imp _and breathing pattern between all cats, the correlations remained high for these parameters when presented in the same graphs (Fig. 5, *panels C and D; p *< 0.01). No such correlations were found between PSR *f*_imp _and PNA amplitude or between PSR *f*_imp _and mean PNA.

### Relationship between tidal volume and inspiratory time during PAV and CPAP

**Figure 6:**
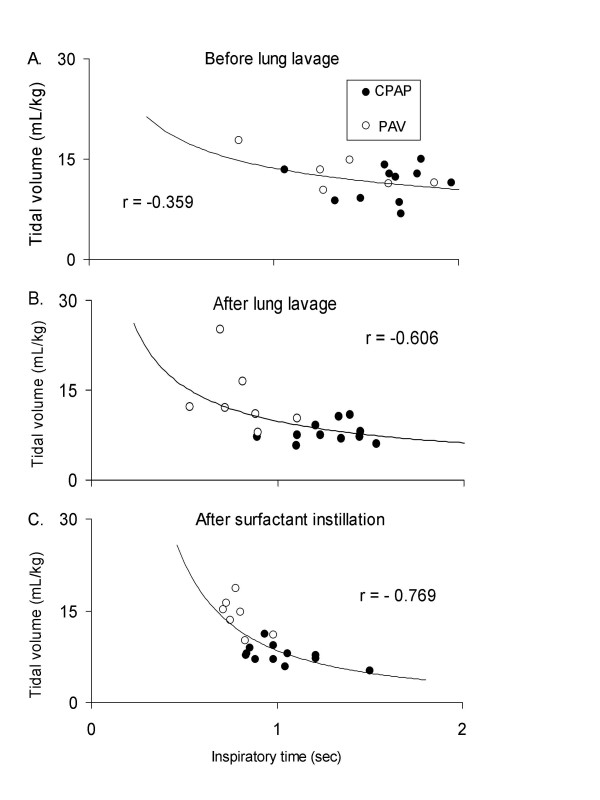
The relationship between tidal volume and inspiratory time during PAV (open circle) and CPAP (filled circle) before lung lavage (A), after lung lavage (B) and after instillation of surfactant (C) (the X-axis is the dependent variable, and Y-axis the independent variable). Each point represents the mean value of 10 breaths. The regression curve (continuous curve) for all mean values is presented with a correlation coefficient (r). A low correlation between tidal volume and inspiratory time is observed in cats with healthy lungs with tidal volume in a normal range (A). The correlation between tidal volume and inspiratory time is increased after surfactant instillation (C).

*Before lung lavage*, a low correlation was observed between tidal volume and inspiratory time, with minor differences in response between CPAP and PAV (Fig. 6, *panel A)*.

*After lung lavage *and *after instillation of surfactant*, there was a high inverse correlation between tidal volume and inspiratory time, during CPAP and PAV, as tidal volume increased during PAV concomitantly with a decrease in inspiratory time (Fig. 6, *panels B and C)*. The highest correlation between tidal volume and inspiratory time was observed after instillation of surfactant (Fig. 6, *panel C*; *r *= -0.769; *p *< 0.01).

## Discussion

The most important finding in this study is that cats maintain their control of breathing during PAV early after instillation of surfactant, with a higher tidal volume and respiratory rate at a lower PNA and esophageal deflection pressure than during CPAP. The time course of and changes in PSR *f*_imp _are in concordance with the changes in the duration of PNA, suggesting that PSR activity is involved in the control of breathing during PAV.

In a recent report it has been shown that both high and low threshold PSRs respond with increased activity after instillation of surfactant in surfactant-depleted, spontaneously breathing cats [16]. In the same study, a decrease in the inspiratory to expiratory time ratio was noted, indicating an increased inhibitory effect of PSRs on the breathing pattern during recovery from RDS. These findings and the reported risk of apnoea after instillation of surfactant in newborn infants with RDS ([17]; meta-analysis of several studies), could have implications for a successful application of patient triggered ventilatory modes, such as PAV, that depend on the spontaneous inspiratory activity.

It has previously been shown that the effects of elastic unloading on the total compliance of the combined lung-respirator system can be predicted with high accuracy during PAV [2]. In a succession of studies, PAV has been investigated in a variety of animal models, with or without lung injury [2,5,6,28], and also in infants with mild RDS [7]. These studies showed that ventilation and oxygenation were improved during PAV in comparison to spontaneous breathing on CPAP [6], and that gas exchange was maintained with lower transpulmonary pressure than during assist control ventilation and intermittent mandatory ventilation [7].

In the present study, lung compliance was low after lung lavage, but increased after instillation of surfactant. Nevertheless, the breathing during CPAP remained rapid and shallow after instillation of surfactant as earlier reported [16]. Respiratory rate and tidal volume were both higher during PAV than during CPAP, resulting in a persistently higher minute volume. After instillation of surfactant, the oxygenation increased and the transpulmonary pressure decreased both during CPAP and PAV, showing an improved gas exchange after surfactant instillation, as reported by other authors [29-32].

The difference in breathing pattern between PAV and CPAP was most clearly evident after lung lavage, with a shorter inspiratory time during PAV than during CPAP, leading to a lower I:E ratio. This could be explained by an earlier and more rapid increase in inspiratory airflow during PAV, whereby maximal tidal volume was reached earlier. In fact, with other techniques for insufflation, Clark and von Euler [8] observed in human and animal studies that when a gain in inspiratory airflow was superimposed on spontaneous breathing, the duration of inspiration was shorter. They ascribed this observation to the volume information supplied by vagal afferents to the respiratory centre, as the inverse relationship between tidal volume and inspiratory time disappeared in vagotomized cats [8].

A similar correlation between tidal volume and inspiratory time was found in the present study, most markedly in the surfactant-depleted animals during CPAP and PAV, as tidal volume increased and inspiratory time decreased (Fig. 6, *panel B and C*). Interestingly, surfactant instillation strengthened the correlation between tidal volume and inspiratory time (Fig. 6, *panel C)*, which might be explained by increased PSR activity after surfactant instillation as earlier reported [16].

The most striking effect of PAV on PNA both after lung lavage and after surfactant instillation was a markedly shorter duration of PNA during PAV than during CPAP (Table 2), illustrating a lower inspiratory effort and reduced work of breathing during elastic unloading [2].

Pack et al [11] described the characteristics of PNA during different ramp inflations in cats with normal lungs, and showed that the immediate increase in PNA with increased airflow was abolished after vagotomy, indicating that PNA is modified by afferent vagal activity. In the present study, there were no differences in tidal volume, PNA (duration and amplitude) or PSR *f*_imp _between CPAP and PAV in cats with normal lungs. After lung lavage a higher tidal volume and respiratory rate, and a higher PSR *f*_imp _and transpulmonary pressure, concomitantly with a lower PNA, were observed during PAV than during CPAP, suggesting the influence of PSR activity on PNA.

In a study of different pressure waveforms with the same airway pressure and tidal volume in cats with healthy lungs, Ehrhardt et al [26] found that the timing of peak PSR activity might influence the PNA. The inspiratory activity (PNA) was more strongly inhibited with a squarewave pressure waveform than with sinusoidal or linear pressure waveforms, with a simultaneously earlier peak PSR *f*_imp _during inspiration and a sustained PSR *f*_imp _at end-inspiration. Similar observations have been made in patients recovering from acute lung injury [33] where the shortest inspiratory rise time significantly reduced the inspiratory activity. These studies also showed that other modes of ventilation might affect the inspiratory activity in a similar way as during elastic unloading with PAV.

The linear correlation between peak PSR *f*_imp _and PNA duration and between time to peak PSR *f*_imp _and PNA duration during PAV in the present study (Fig. 5) indicates the importance of the timing of PSR activity for the control of breathing during this ventilatory mode. Furthermore, it suggests that the strength of PNA, in terms of mean PNA and PNA amplitude is probably more dependent on other factors than PSR activity, such as the chemical drive of a rise in P_CO2 _and a low pH, or other peripheral receptors. After lung lavage, respiratory rate was higher during PAV than during CPAP and I:E ratio was lower during PAV than during CPAP, which shows that the inspiratory inhibitory reflex seems to be activated earlier during PAV than during CPAP.

During PAV, the increase in PNA occurring after instillation of surfactant could be due to a reduction in PSR *f*_imp _in response to a lower transpulmonary pressure, as both tidal volume and arterial PaCO_2 _remained unaltered (Fig. 4). Thus, both changes in airflow [11] and in transpulmonary pressure [20] seem to influence the breathing effort during PAV, and more so in the surfactant-depleted cat.

In the present study, elastic unloading with PAV was applied only during inspiration. The release of a high positive airway pressure at end-inspiration could potentially cause a momentary change in the stretching of the lung and consequently altered PSR activity. Such a change in esophageal deflection pressure at end-inspiration was observed after lung lavage, as illustrated by the rebound of esophageal pressure (loss of transmitted inspiratory pressure from the ventilator) seen in Figure 4, but without a concomitant change in PSR response or PNA. In fact, Cross et al [10] reported that inflations near the end of inspiration induced no PNA response, thus demonstrating that volume information in the third phase of inspiration played only a minor role in modulating the ongoing breathing effort. In the present study, the immediate decrease in positive airway pressure and the immediate increase in esophageal pressure at end-inspiration during PAV indicate that the PSRs were subjected to approximately the same transpulmonary pressure during that phase (Fig. 4). The combination of increased airway pressure (Fig. 2) and reduced esophageal deflection pressure during PAV (Table 1), compared to that during CPAP, could give rise to differences in stimulation of the PSRs. However, PSR *f*_imp _in relation to transpulmonary pressure did not differ between CPAP and PAV either before or after lung lavage, or after instillation of surfactant (Table 2), implying that transpulmonary pressure elicited a similar response from the PSRs during CPAP and PAV. Although PSRs were exposed to higher transpulmonary pressures during PAV than during CPAP both after surfactant-depletion and instillation of surfactant (Table 1), apnea was not elicited in any of the cats studied.

Irregularities of breathing as observed in surfactant-depleted human neonates [31] were noted in the present study after lung lavage and surfactant instillation, both during CPAP and PAV. But only after lung lavage was the C.V. for respiratory rate higher with PAV than with CPAP, a difference that was mainly due to a higher C.V. for expiratory time. These irregularities did not have any influence on the effects of PAV, nor did they alter the increase in tidal volume during PAV in comparison to CPAP.

## Conclusion

In surfactant-depleted cats, the PSR activity and the control of breathing are maintained early after surfactant treatment during CPAP and PAV, but with an increased depth and a higher rate of breathing and a lower breathing effort during PAV than during CPAP.

## List of abbreviations

**C.V**., coefficient of variation

**CPAP**, continuous positive airway pressure

***f*_imp_**, instantaneous impulse frequency

**I:E ratio**, inspiratory to expiratory time ratio

**PAV**, proportional assist ventilation

**PNA**, phrenic nerve activity

**PSR**, slowly adapting pulmonary stretch receptor

**RDS**, respiratory distress syndrome

## Competing interests

The author(s) declare that they have no competing interests.

## Authors' contributions

RS participated in designing the study, was responsible in the preparation and care of the animals and for the neuro-physiological preparation, for the acquisition and analysis of the data and for writing the manuscript. ERF participated in the preparation of the animals, was involved in the acquisition of the data, and revising the manuscript. AJ participated in the design of the study, was responsible for the preparation of the animals and for the neuro-physiological recordings, and for revising the manuscript. AS participated in designing the study, was responsible for the theoretical basis of the ventilatory mode, as well as for the ventilatory settings, and provided new methods for the data acquisition and analysis, helped to interpret the data and revised the manuscript. PS provided the technical knowledge for the ventilatory methods studied, and was responsible for the on-line data acquisition from the ventilator, and revised the part of the manuscript concerning the technical application of PAV. GS conceived of the study and its design, performed the lavage, helped to interpret the data, and revised the manuscript. All authors read and approved of the final manuscript.
